# *Rad9a* is required for spermatogonia differentiation in mice

**DOI:** 10.18632/oncotarget.13405

**Published:** 2016-11-16

**Authors:** Lin Huang, Zhen-Bo Wang, Shu-Tao Qi, Xue-Shan Ma, Qiu-Xia Liang, Guo Lei, Tie-Gang Meng, Li-Feng Liang, Ye-Xin Xian, Yi Hou, Xiao-Fang Sun, Yong Zhao, Wei-Hua Wang, Qing-Yuan Sun

**Affiliations:** ^1^ Key Laboratory of Major Obstetrics Diseases of Guangdong Province, Key Laboratory of Reproduction and Genetics of Guangdong Higher Education Institutes, The Third Hospital Affiliated to Guangzhou Medical University, Guangdong, China; ^2^ State Key Laboratory of Stem Cell and Reproductive Biology, Institute of Zoology, Chinese Academy of Sciences, Beijing, China; ^3^ University of the Chinese Academy of Sciences, Beijing, China; ^4^ State Key Laboratory of Biomembrane, Institute of Zoology, Chinese Academy of Sciences, Beijing, China; ^5^ Houston Fertility Institute/Houston Fertility Laboratory, Houston, Texas, USA

**Keywords:** RAD9A, Vasa, spermatogenesis, Sertoli cells, differentiation

## Abstract

Spermatogenesis in testes requires precise spermatogonia differentiation. Spermatocytes lacking the *Rad9a* gene are arrested in pachytene prophase, implying a possible role for *RAD9A* in spermatogonia differentiation. However, numerous *RAD9A*-positive pachytene spermatocytes are still observed in mouse testes following *Rad9a* excision using the *Stra8-Cre* system, and it is unclear whether *Rad9a* deletion in spermatogonia interrupts differentiation. Here, we generated a mouse model in which *Rad9a* was specifically deleted in spermatogonial stem cells (SSCs) using Cre recombinase expression driven by the germ cell-specific *Vasa* promoter. Adult *Rad9a*-null male mice were infertile as a result of completely blocked spermatogonia differentiation. No early spermatocytes were detected in mutant testicular cords of 9-day-old mice. Mutant spermatogonia were prone to apoptosis, although proliferation rates were unaffected. *Rad9a* deletion also resulted in malformation of seminiferous tubules, in which cells assembled irregularly into clusters, and malformation led to testicular cord disruption. Our findings suggest that *Rad9a* is indispensable for spermatogonia differentiation and testicular development in mice.

## INTRODUCTION

Mammalian spermatogenesis is a highly organized process that requires precise progressive differentiation of spermatogonia. Both somatic and germ cell-specific factors play critical roles in this process. As the primary somatic cells in seminiferous epithelium, Sertoli cells interact directly with germ cells at different stages, assuring normal spermatogonia differentiation [1]. Sertoli cell-specific factors are necessary for spermatogonia differentiation and tesitcular cord maintenance [2–4]. However, few studies have focused on the roles of germ cell-specific factors in these processes.

Mouse *Rad9a* is an evolutionarily conserved gene that helps maintain genome integrity [5]. RAD9A functions as part of the heterotrimeric 9-1-1 complex composed of RAD9A, RAD1 and HUS1 [6]. RAD9A participates in the cellular response to exogenous DNA damage via the ataxia telangiectasia and Rad3-related (ATR) and ataxia telangiectasia mutated (ATM) signaling pathways [7–9]. It also influences DNA repair directly by physically interacting with proteins involved in DNA replication and homologous recombination [10–17]. In addition to its well-known function in DNA damage repair, mammalian RAD9A regulates cell cycle checkpoints and apoptosis. *In vitro*, *Rad9a* knockdown increased spontaneous, as well as topoisomerase poison-induced death, spontaneous chromosomal aberrations, and radio-resistant DNA synthesis [6, 18–21]. RAD9A deficiency also impairs cell proliferation, migration and invasion [21, 22]. *In vivo*, embryos with homozygous *Rad9a* deletions died nine days post coitum [6]. While *Rad9a* null embryonic stem cells are viable, mouse embryonic fibroblasts devoid of *Rad9a* are not [6].

In a recent report, *Rad9a* was conditionally knocked out in undifferentiated spermatogonia in a mouse model via Cre recombinase expressed under the *Stra8* promoter (*Stra8-Cre*) [23]. Spermatogenesis was only interrupted at the late zygotene or early pachytene stage of meiotic prophase I, indicating that Rad9a deletion did not fully inhibit spermatogonia differentiation. This may have been due to the fact that the deletion was carried out in later spermatogonia. Moreover, deletion of *Rad9a* via *Stra8-Cre* may not be entirely effective, as spermatocytes in mutant testes can escape floxed allele excision [23]. To investigate whether RAD9A functional disruption wholly blocks spermatogonia differentiation, and to assess the impacts of this disruption on testicular cord development, we generated mice in which *Rad9a* was conditionally deleted in spermatogonial stem cells via Cre recombinase driven by the *Vasa* promoter. While mutant spermatogonia were viable and proliferated normally, differentiation was completely blocked. Additionally, *Rad9a* deletion led to malformation of testicular cords. Our results show for the first time that RAD9A is indispensable for spermatogonia differentiation and testicular cord maintenance.

## RESULTS

### *Rad9a* deletion in spermatogonial stem cells leads to male infertility

**Figure 1:**
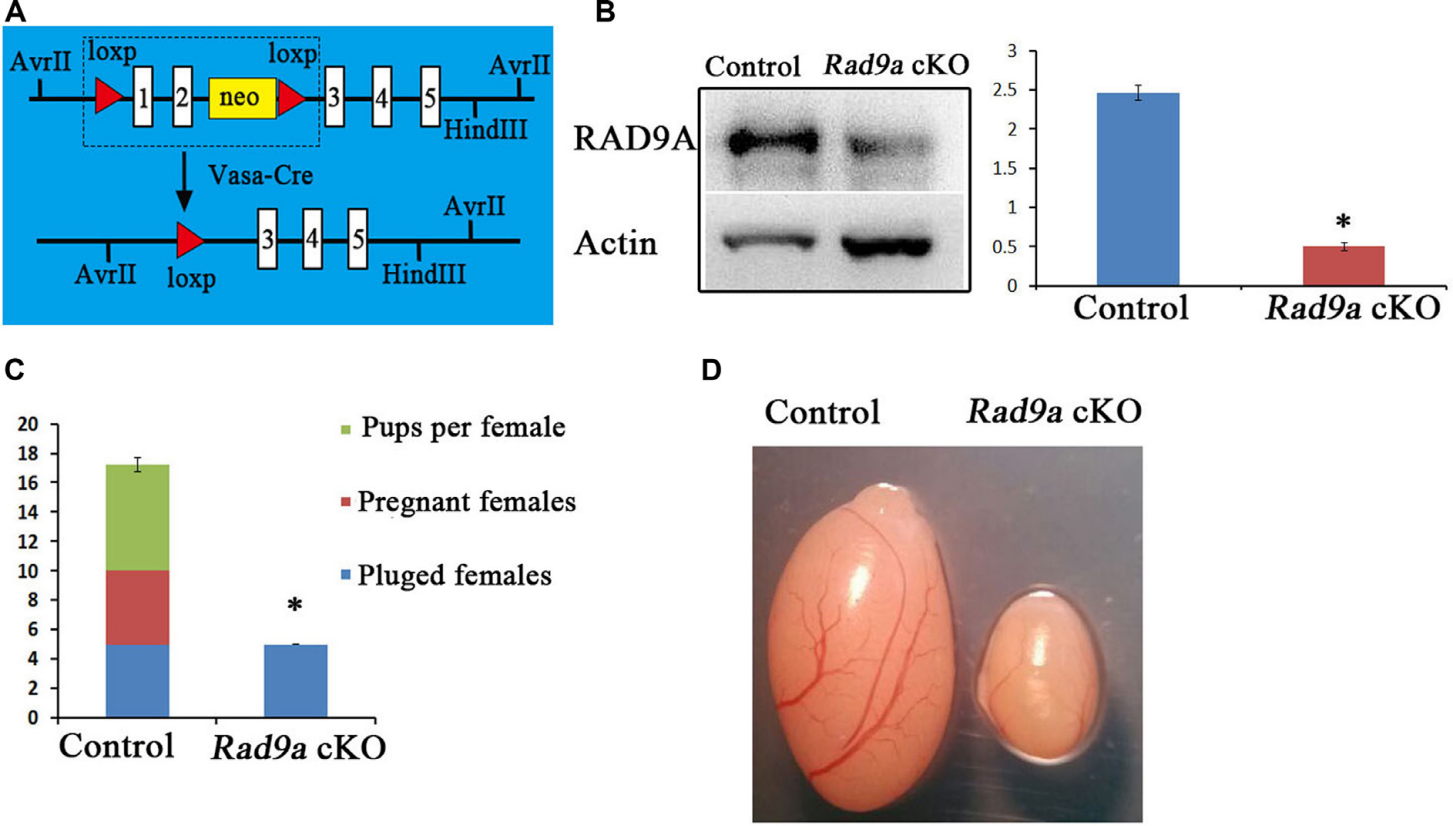
Conditional Rad9a deletion in SSCs via Vasa-Cre Schematic of deletion of the floxed *Rad9a* exon1 and exon2 via *Vasa*-Cre mediated recombination (**A**) Western blot analysis of RAD9A in P9 testis extracts from both wild type and *Rad9a* cKO mice (**B**) Results were normalized to the actin loading control. The experiment was repeated three times. Fertility test (**C**) Plugged females became pregnant and gave birth to pups after crossing with *Rad9a*^F/F^ control males, whereas none of the plugged females were pregnant after crossing with *Rad9a* cKO males. Testes from *Rad9a*^F/F^ control males and *Rad9a* cKO males at six months (**D**) Testis size in *Rad9a* cKO mice was sharply reduced compared with that of control mice. *P < 0.01.

Because conventional *Rad9a* deletion results in embryonic lethality, we constructed a mouse model with *Rad9a* deleted specifically in spermatogonia [6]. We crossed *Rad9a^F/F^* mice with *Vasa-Cre* transgenic mice, in which Cre begins to be expressed in primordial germ cells (PGC), to conditionally delete *Rad9a* in offspring PGCs (hereafter referred to as *Rad9a* cKO mice) (Figure 1A) [6, 24]. Western blotting results showed that RAD9A levels were sharply reduced in testis tissues from *Rad9a* cKO male mice as compared to *Rad9a^F/F^* mice. Residual RAD9A observed in knockout mice testes may come from Sertoli cells [23].

We assessed the effects of RAD9A deletion in PGCs on the reproductive performance of mutant male mice. None of the plugged female mice became pregnant after crossing with mutant males and no pups were produced (Figure 1C). In comparison, almost all female mice with vaginal plugs that mated with wild type mice became pregnant and produced seven pups each on average. We observed that the testes of *Rad9a* cKO male mice at six months of age were much smaller than those of *Rad9a^F/F^* mice (Figure 1D).

### Mutant testes develop disorganized testicular cords

**Figure 2:**
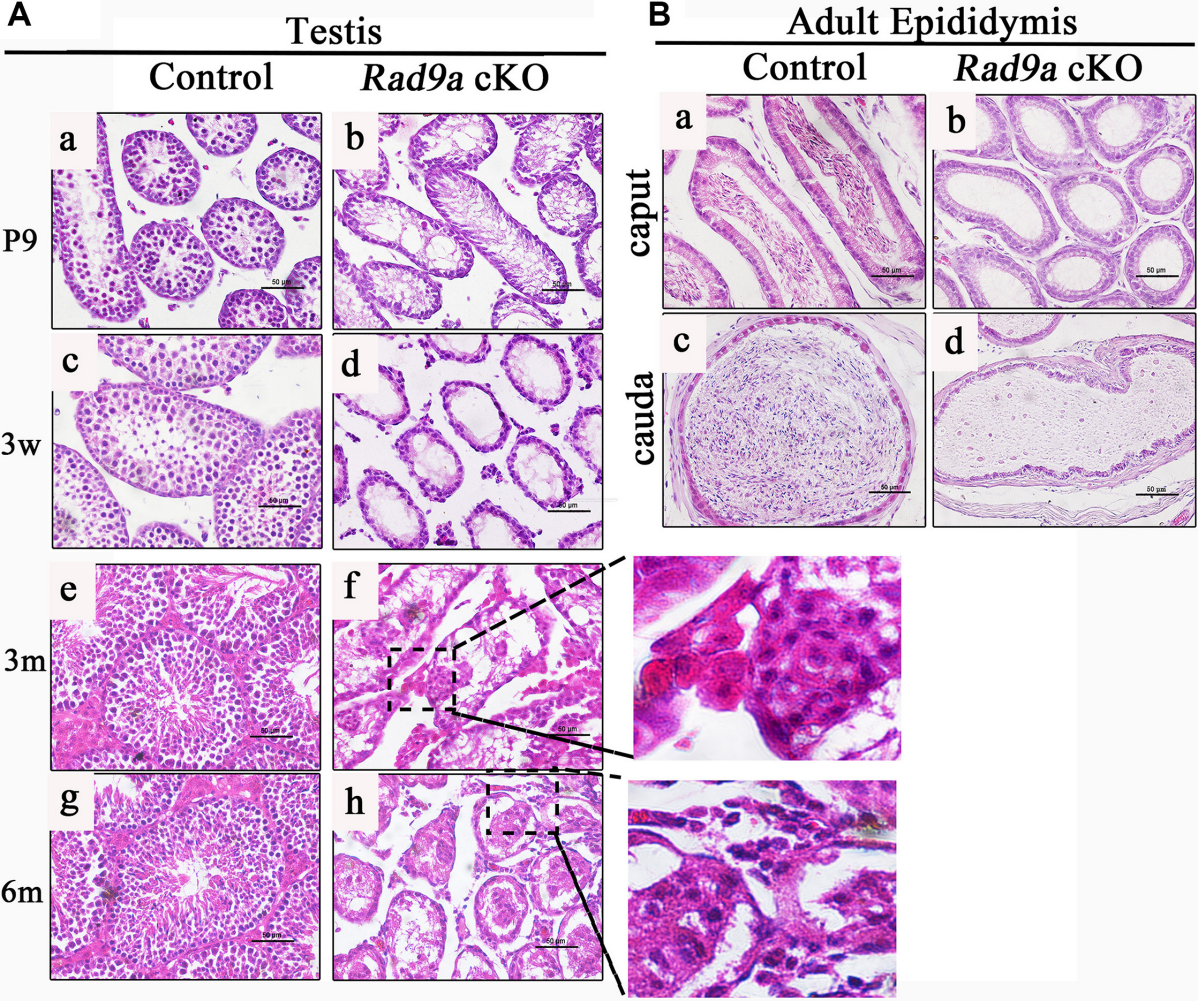
H&E staining of test is and epididymis tissues Comparison of testicular cords from *Rad9a*^F/F^ control males and *Rad9a* cKO males (**A**) In contrast to control testicular cords (a, c, e, g), *Rad9a* cKO mouse testes became vacant at as early as nine days of age (**B**). The difference became more significant at three weeks (d). Testes of *Rad9a* cKO adult males developed disorganized testicular cords with malformed cell clusters (f). Disorganized testicular cords in mutant testes collapsed (h). Both the caput and cauda epididymides of control adult male mice were full of mature sperm (a, c), but were vacant in adult *Rad9a* cKO males (b, d) b.

To assess the time course of abnormal testicular development, we compared testis histological morphology from *Rad9a^F/F^* and *Rad9a* cKO male mice at postnatal day nine (P9), three weeks, three months and six months of age. As previously described [25], early spermatocytes began to appear in the testes of wild type mice at P9 (Figure 2Aa). Round spermatids were observed in *Rad9a^F/F^* male mouse testes at three weeks (Figure 2Ac). In adulthood (three and six months), spermatogonia, spermatocytes, round spermatids, and condensed mature spermatids were arranged regularly by Sertoli cells in *Rad9a^F/F^* male mouse testicular cords (Figure 2Ae and 2Ag). In comparison, *Rad9a* cKO male mouse testicular cords became vacant as early as P9 (Figure 2Ab). Differences between control and mutant testicular cords became more significant at three weeks of age (Figure 2Ad). Only a single layer of cells remained around the basal membrane in most conditional knockout testicular cords, in contrast to that of control mice. Until three months of age, mutant testicular cords were extremely disorganized (Figure 2Af). Irregularly assembled cell clusters were observed in most cords, and only basal membrane was residual in parts of the abnormal cords. No basal membrane was present in some cases. Phenotypes were more severe in *Rad9a* cKO male mice testes at six months (Figure 2Ah). Some mutant testicular cords appeared to collapse and some cells penetrated testicular cords into the interstitum of the testes.

To confirm the testis phenotypes, we examined adult epididymides from both genotypes. Both the caput and cauda of control epididymides were full of mature condensed sperm (Figure 2Ba and 2Bc). However, the caput epididymides of knockout mice were vacant, without any cells (Figure 1Bb). There was also no sperm in mutant cauda epididymides, which were filled with lipid fluid (Figure 2Bd). These data indicated that conditional *Rad9a* deletion in spermatogonia completely blocked spermatogenisis, led to disorganized cell clusters in the testicular cords, and finally disaggregated the testicular cords.

### Malformed cell clusters in mutant testicular cords mainly consisted of Sertoli cells

**Figure 3:**
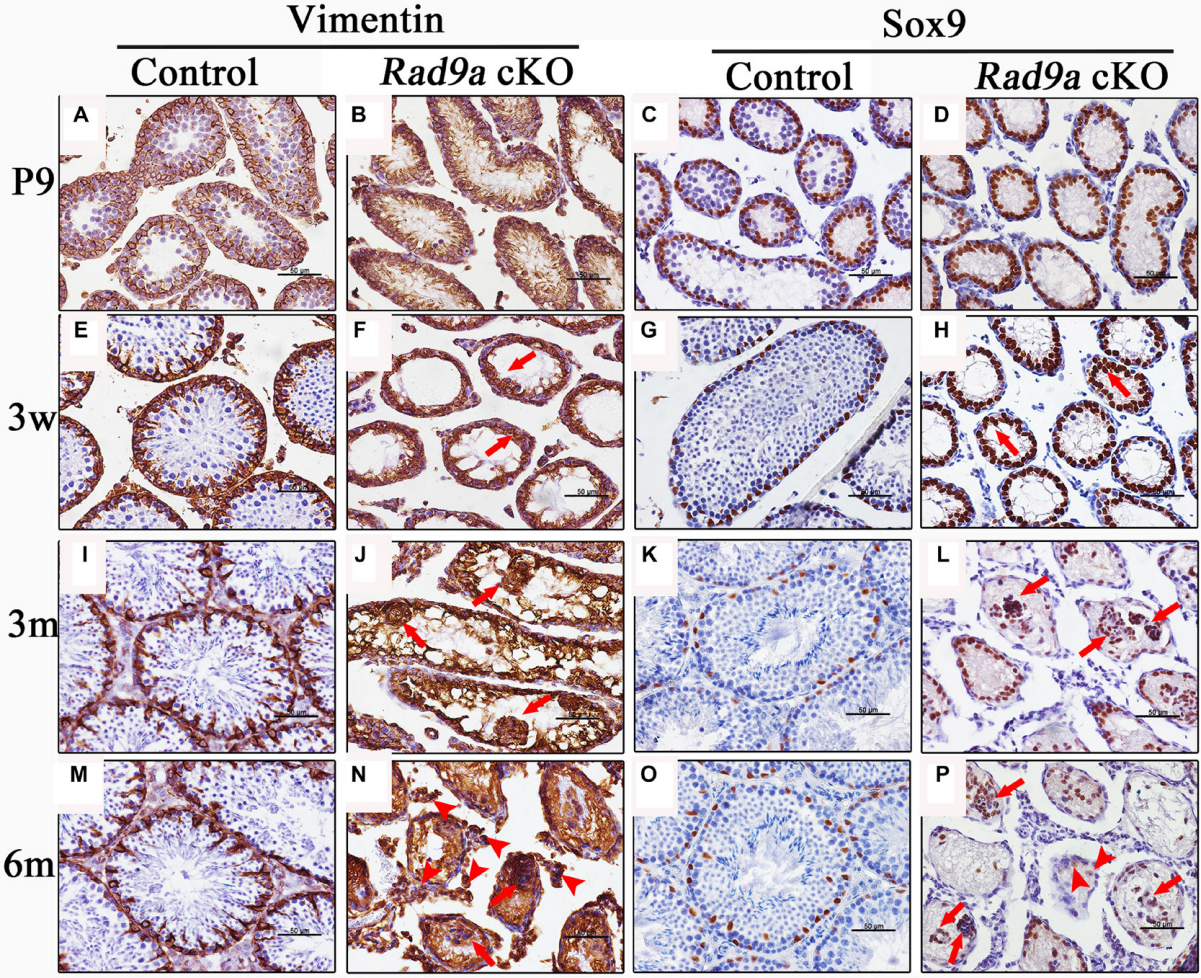
Malformed cell clusters mainly consisted of Sertoli cells *Rad9a*^F/F^ control and *Rad9a* cKO testes at different ages were immunostained with two Sertoli cell markers: Vimentin and Sox9. Nuclei were stained blue with hematoxylin. Vimentin accumulated at the junction between germ cells and Sertoli cells in the control testes at P9. Vimentin presented as a regularly radial pattern from three weeks to adulthood (**A**, **E**, **I**, and **M)**. Vimentin was evenly dispersed throughout Sertoli cells in *Rad9a* cKO testes at P9, and abnormal Vimentin distribution became increasingly apparent from three weeks to adult hood (**B**, **F**, **J**, and **N)**. In control testes, Sox9-positive cells began to migrate to the basal membrane at P9, and located to the basal membrane (**C**, **G**, **K**, and **O)**. In *Rad9a* cKO testes, Sox9-positive cells migrated to the lumen from P9, and finally assembled into malformed cell clusters with few Sox9-negative cells (**D**, **H**, **L**, and **P)**.

To investigate the origins of malformed cell clusters observed in mutant testicular cords, we determined cell types using molecular markers. Intermediate filaments in Sertoli cells of postnatal mice are comprised of Vimentin, which is also a Sertoli cell tumor marker [26, 27]. Vimentin accumulated at the boundary between Sertoli and germ cells in control mouse testes at P9. (Figure 3A), and its accumulation was more significant at three weeks (Figure 3E). At adulthood, Vimentin staining presented as a regularly radial pattern in control testicular cords (Figure 3I and 3M). In contrast, Vimentin was evenly dispersed throughout mutant mouse Sertoli cells at P9 (Figure 3B). At three weeks, disorganized Vimentin distributions appeared in knockout mouse testicular cords (Figure 3F, arrows). At three months, Vimentin staining accumulated around malformed cell clusters in the mutant testicular cords (Figure 3J, arrows). In mutant mouse testes at six months, Vimentin immunostaining was observed outside the testicular cords (Figure 3N, arrowheads), as well as around malformed cell clusters (Figure 3N, arrows).

These findings indicated that malformed cell clusters in mutant testes may consist primarily of Sertoli cells. To confirm this, we compared Sox9 immunostaining, another Sertoli cell marker, in the testes of both genotypes. Most Sertoli cell nuclei localized to the basal membrane as early as P9 in wild type testes (Figure 3C), and staining remained consistent from three weeks to adulthood (Figure 3G, and 3O). However, Sertoli cell nuclei in mutant testes at P9 were poorly orientated to the basal membrane (Figure 3D), and some began to migrate toward the lumen of mutant testicular cords at three weeks (Figure 3H, arrows). Migrating Sertoli cell nuclei assembled to form clusters in adult testicular cords (Figure 3L and 3P, arrows). We observed some cells without Sox9 staining in the malformed cell clusters (Figure 3L and 3P, arrows), as well as cells with weakened Sox9 signal among cells separated from disrupted testicular cords (Figure 3P, arrowheads).

### Spermatogonia differentiation is blocked in mutant testes

**Figure 4:**
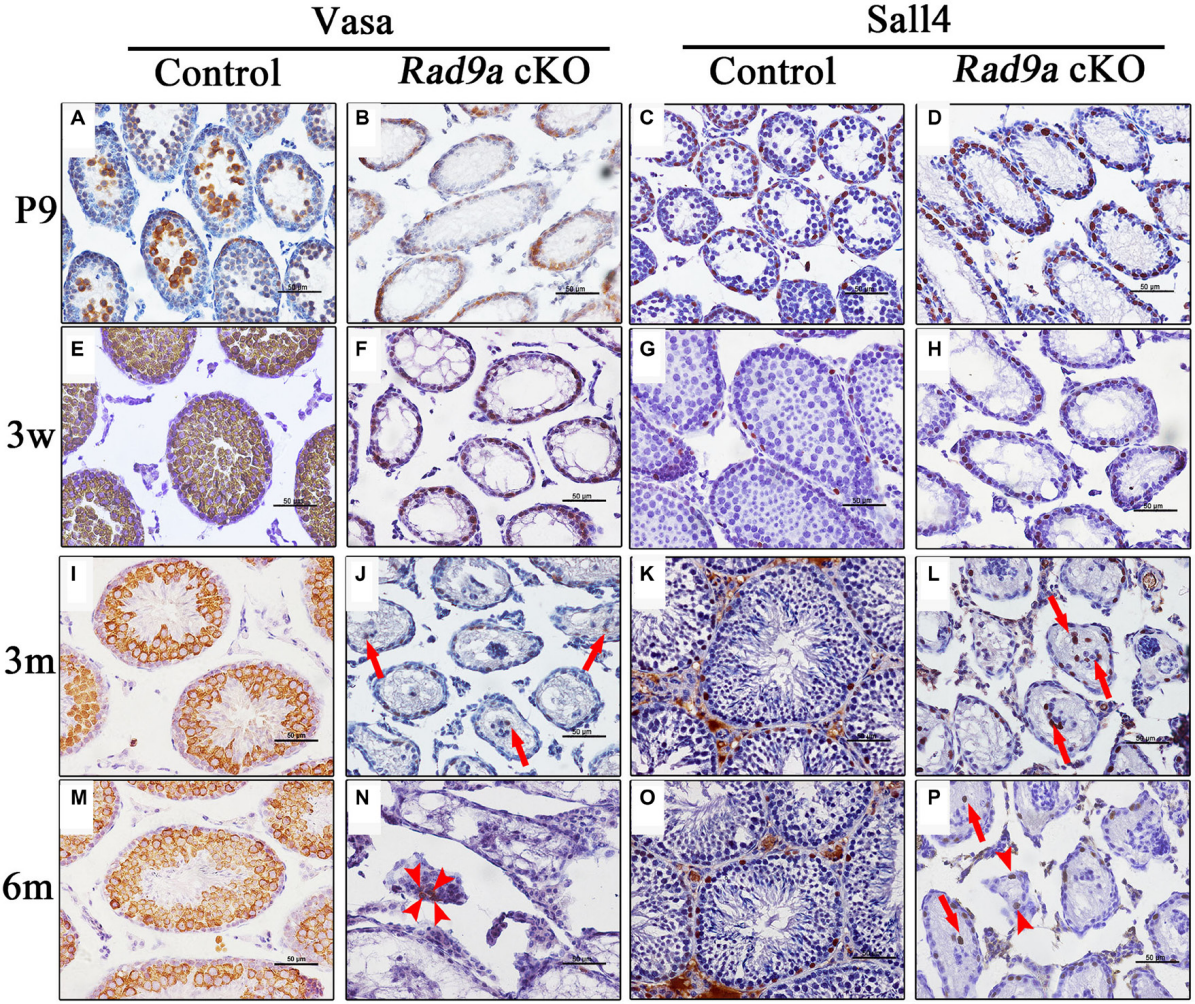
Spermatogonia differentiation was inhibited in mutant testes Representative seminiferous tubule images at P9 (**A**–**D)**, three weeks (**E**–**H**), three months (**I**–**L)**. and six months of age (**M**–**P)**. with staining of MVH (a germ cell marker) and Sall4 (a spermatogonia marker, brown). Nuclei are stained blue with hematoxylin.

Vasa (also named MVH) is a germ cell-specific marker in testis, and is expressed throughout the spermatogonium-to-round spermatid development process. We observed moderately Vasa-positive spermatogonia at the basal membrane, as well as early spermatocytes with strong Vasa signal in the lumen of wild type testicular cords at P9 (Figure 4A). Along with spermatogonia and spermatocytes, Vasa-positive round spermatids appeared at three weeks in control testes (Figure 4E), after which Vasa-positive germ cell staining patterns remained consistent (Figure 4I and 4M). In contrast, we observed Vasa-positive spermatogonia only at the basal membrane of mutant testicular cords at P9 (Figure 4B). No Vasa-positive spermatocytes were observed in the lumen of mutant testicular cords, except for moderately Vasa-positive spermatogonia at the basal membrane at three weeks (Figure 4E). We observed only sporadically Vasa-positive germ cells in adult mutant testicular cords (Figure 4J and 4N), some at the basal membrane, and some in the lumen, where they formed disorganized clusters with Sertoli cells (Figure 4J, arrows). We also detected a few Vasa-positive cells among the interstitial cells, indicating leakage of cells from disrupted testicular cords to the interstitium (Figure 4N, arrowheads).

These data implied that spermatogonia development might also be disrupted following *Rad9a* deletion. We traced spermatogonia fates via immunostaining with pluripotency factor Sall4, which is implicated in stem cell maintenance and restricted to gonocytes and undifferentiated spermatogonia [28]. All Sall4-positive cells were restricted at the basal membrane from P9 to adulthood in control testes (Figure 4C, and 4O). All Sall4-positive cells were also located at the basal membrane in mutant testicular cords at P9 (Figure 4D), but some Sall4-positive spermatogonia in mutant testes began to migrate away from the basal membrane at three weeks (Figure 4H). However, we still observed Sall4-positive spermatogonia at the basal membranes of some mutant testicular cords at adulthood (Figure 4L and 4P). Some Sall4-positive cells had migrated to the lumen of mutant testicular cords, assembling with Sall4-negative cells at adulthood (Figure 4L and 4P, arrows). As indicated by Vasa immunostaining, very few Sall4-positive cells embedded with other cells moved from disorganized testicular cords to the interstitium (Figure 4P, arrowheads). These data indicated that *Rad9a* deletion blocked spermatogonia differentiation, but spermatogonia nonetheless appeared viable.

### Mutant spermatogonia proliferate normally, but are prone to apoptosis

**Figure 5:**
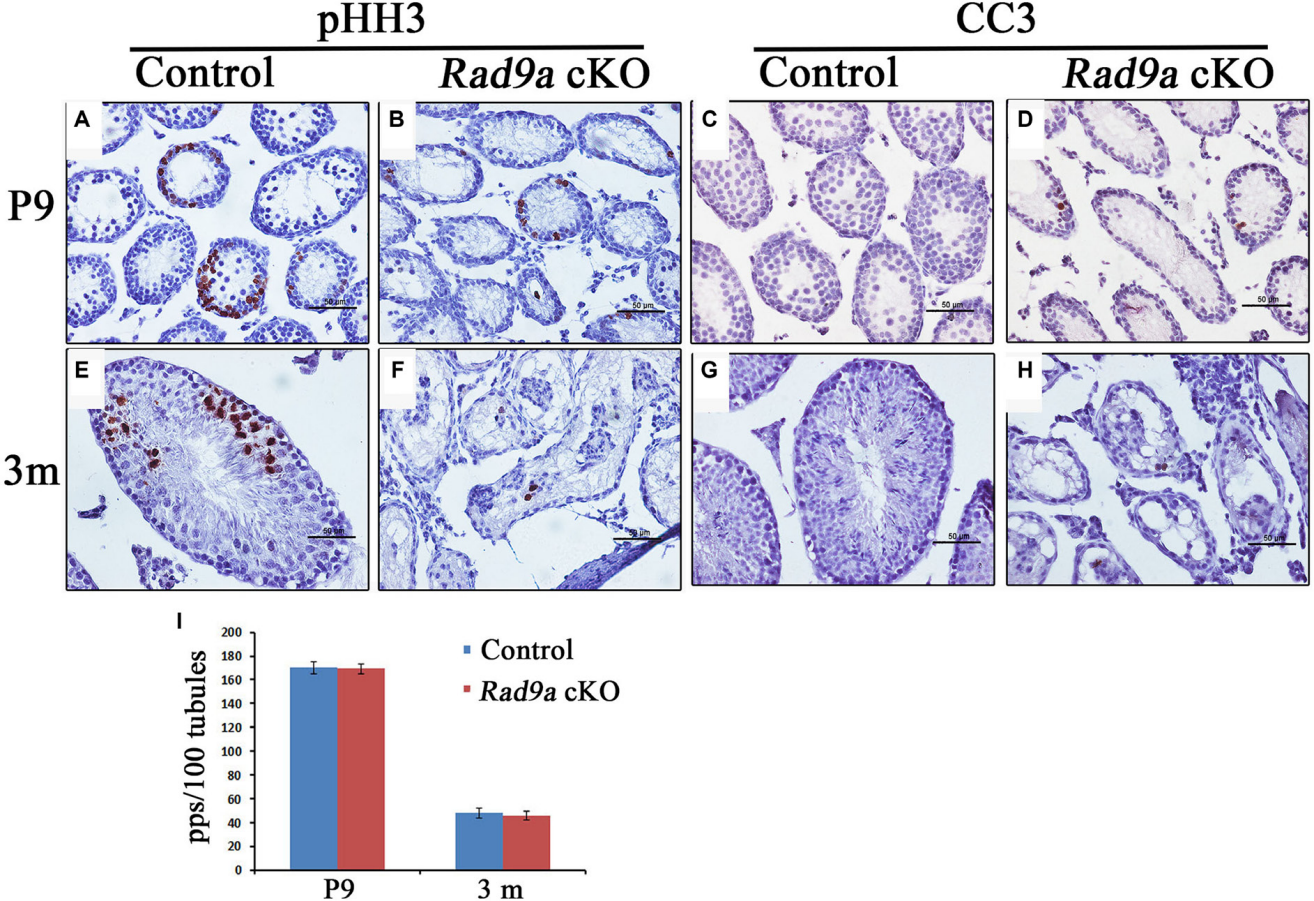
Mutant spermatogonia were prone to apoptosis More pHH3-positive cells can be seen in *Rad9a*^F/F^ control testes compared to *Rad9a* cKO testes at P9 and three months (**A**, **B**, **E**, and **F)**. The number of pHH3-positive spermatogonia was the same for both genotypes (**I)**. Mutant spermatogonia were prone to apoptosis at P9 and three months (**C**, **D**, **G**, and **H)**. pps, pHH3-positive spermatogonia.

We assessed mutant spermatogonia proliferation rates through immunohistochemical staining with the mitosis marker, phosphorylated histone H3 (pHH3). We observed strong pHH3 signals at P9 in control testes (Figure 5A), with fewer pHH3-positive cells in *Rad9a* cKO male mouse testicular cords (Figure 5B). Control testicular cords showed increased pHH3-positive germ cells at adulthood (Figure 5E), but we observed only sporadic pHH3-positive cells in mutant testicular cords (Figure 5F). pHH3-positive germ cells in control testis included both spermatogonia and spermatocytes. No significant difference was found in the number of pHH3-positive spermatogonia between control and mutant testicular cords (Figure 5I), indicating that mutant and wild type spermatogonia proliferated at the same rate.

RAD9A inhibits apoptosis, and cells in *Rad9a* null embryos were prone to spontaneous apoptosis [29, 30]. To test whether *Rad9a* deletion also promotes apoptosis in spermatogonia, we stained tissues for cleaved caspase 3 (CC3). Almost no CC3-positive germ cells were observed in control testicular cords at P9 or adulthood (Figure 5C and 5G). In contrast, we frequently observed CC3-positive germ cells in conditional knockout mouse testicular cords at both P9 and adulthood (Figure 5D and 5H). CC3-positive germ cells included both early spermatocytes and spermatogonia. These results suggest that RAD9A inhibits spermatogonia apoptosis, although spermatogonia devoid of RAD9A proliferate normally.

## DISCUSSION

The 9-1-1 complex is a critical participant in several cellular processes, including DNA damage repair, homologous recombination, and cell cycle checkpoint control in both mitotic and meiotic cells [31–33]. However, the function of mammalian RAD9A in spermatogonia differentiation had not yet been elucidated. In this study, we constructed and characterized a mouse model with *Rad9a* deleted in spermatogonial stem cells (SSCs). Mutant males developed normally with typical reproductive behavior. However, none produced pups, apparently due to reduced testis size. Subsequent histological analyses of conditional knockout mouse testes indicated that mutant testicular cords were vacant as early as P9, leaving only Sertoli cells and spermatogonia at the basal membrane. These results were confirmed by Sertoli and germ cell marker immunostaining. We validated that no mutant spermatogonia entered meiosis. Thus, spermatogonia differentiation was completely blocked in mutant males. When Vasileva, *et al*. [23] deleted *Rad9a* in spermatogonia via *Stra8-Cre*, spermatogenesis was interrupted at the late zygotene or early pachytene stage of meiotic prophase I, and prophase spermatocytes survived normally in mutant testicular cords at adulthood. Differences between these results and those of the present study may result from knockout efficiency variations between the two mouse models. Many RAD9A-positive pachytene spermatocytes are still observed in testis when the *Stra8-Cre* knockout system is employed. This suggests that spermatocytes and spermatogonia might escape Cre excision [23]. In contrast, Cre excision in our study was much more effective in SSCs. As *Rad9a* null spermatogonia remained alive and proliferated normally in our study, blocked spermatogonia differentiation may not be attributed to defective DNA damage repair or other known RAD9A functions. Consistent with our results, *Rad9a* null mouse embryonic stem cells are reportedly viable and proliferate normally, while mouse embryonic fibroblasts with the same genotype cannot be established [6]. These data imply a currently unknown mechanism by which RAD9A protects differentiated cells against apoptosis. More studies are needed to elucidate this mechanism.

We observed malformed testicular cords in mutant testes at adulthood, and cells assembled irregularly to form disorganized cell clusters in mutant testicular cords. Some tubular architecture was also disrupted, and cells broke away into the interstitia of mutant testes. Interestingly, we observed Sox9- and Sall4-positive cells among these interstitium-localized cells. This was a novel phenotype, although it was relatively mild compared to disruption of testicular cords by Sertoli cell-specific factor dysfunction [1–4]. Testicular cord architecture remains intact following *Stra8-Cre*-mediated *Rad9a* deletion, although spermatogenesis is interrupted. This difference compared to our system may result from the fact that pachytene stage spermatocytes are present in adult male mutant testes in the *Stra8-Cre* system. This indicates that an intact blood-testis barrier has been constructed in mutant testicular cords. In contrast, no early spermatocytes were observed in mutant testes at P9 in our study. Our data suggest that spermatogonia differentiation blocked by *Rad9a* deletion may destroy junctions, such as the blood-testis barrier, and distort testicular construction. More detailed studies are needed to define which types of junctions are destroyed, and to elucidate the mechanisms underlying testicular cord malformation.

In conclusion, our findings revealed that RAD9A plays a key role in spermatogonia differentiation. *Rad9a* null spermatogonia failed to differentiate into spermatocytes, and testicular cord malformation was observed in mutant mouse testes, which led to complete male infertility.

## MATERIALS AND METHODS

### Mice

*Rad9a^flox/flox^* mice (JAX Lab, referred to as *Rad9a^F/F^*) were maintained with a mixed genomic background of 129S4/SvJae and C57/BL6, and *Vasa-Cre* mice were maintained with a C57/BL6 genomic background [6, 24]. Mice with conditional *Rad9a* deletion (referred to as *Rad9a* cKO mice) were generated by crossing *Rad9a^F/F^* mice with *Vasa-Cre* mice. Mice were housed under controlled environmental conditions with free access to water and food, and 12-hour alternating light/dark cycles. Animal care and handling were conducted according to the guidelines of the Animal Research Committee of the Institute of Zoology, Chinese Academy of Sciences, China.

### Fertility assay

To assess the fertility of *Rad9a^F/F^* and *Rad9a* cKO males, we selected five healthy two-month-old males for each genotype and mated them to five six-week-old C57/BL6 females. Females with vaginal plugs after crossing were maintained separately. At about day 25 after checking vaginal plugs, females were sacrificed and the number of pregnant females and living pups were recorded.

### Histological analysis and immunochemistry

Mice were euthanized via cervical dislocation and testes were dissected and fixed immediately in 4% paraformaldehyde overnight at 4°C. Tissues were dehydrated in gradient ethanol, made transparent in xylen, and embedded in paraffin. Embedded tissues were cut into 5 μm sections, which were deparaffinized and rehydrated for histological analysis and immunochemistry. Hematoxylin and eosin (H&E) staining was performed for histological observation. For immunostaining, after antigen retrieval in 10 mM sodium citrate buffer, the endogenous peroxidase was cleaned up by incubating slides in 3% H_2_O_2_. Sections were then blocked with 5% bovine serum albumin (BSA) and incubated with primary antibody at 4°C overnight. After incubation in secondary antibody for 1 h, signals were detected using the Vectastain ABC kit (Vector Laboratories, Burlingame, CA, USA). The following primary antibodies were used in this study: rabbit anti-Sox9 (Abcam, Cambridge, UK; ab3697), rabbit anti-Vasa (Abcam, ab13840), rabbit anti-phospho-HH3 (Abcam, ab5176), mouse anti-Sall4 (Santa Cruz, CA, USA; sc101147), rabbit anti-Vimentin (Zhongshan, Beijing, China; za0511), and rabbit anti-cleaved caspase 3 (CC3) (Cell Signaling Technology, Beverly, MA, USA; 9661).

### Western blot analysis

Mice were sacrificed at about nine days of age, and testes were separated and lysed in radio immunoprecipitation assay lysis buffer containing Complete Mini Protease Inhibitor Cocktail Tablets (Roche). Samples were mixed with SDS sample buffer and boiled for 5 min at 100°C for SDS-PAGE. Western blotting was performed as described previously [34]. Proteins were electrophoresed under reducing conditions in 12% SDS-PAGE gels and transferred to nitrocellulose membranes. Blots were blocked in 5% BSA and incubated overnight at 4°C with primary antibody, followed by incubation with secondary antibody for 1 h at room temperature. Signals were detected using the ECL western blotting detection system. The following primary antibodies were used in this study: mouse anti-RAD9A (Zen Biosciences, Chengdu, China; 200784), and rabbit anti-GAPDH (CST, 5174) according to the manufacturer’s instructions. Secondary antibodies were purchased from ZhongShan Golden Bridge Biotechnology Co., Ltd (Beijing, China).

### Statistical analysis

All experiments were repeated at least three times. For histological analysis and immunochemistry, one representative image of the results from several independent experiments was selected for presentation. For comparisons, means and standard deviations were calculated, and the difference between two groups was analyzed using Student’s *t*-test. Differences were considered statistically significant if *P* < 0.05.
